# Evaluating Heterogeneity of Primary Lung Tumor Using Clinical Routine Magnetic Resonance Imaging and a Tumor Heterogeneity Index

**DOI:** 10.3389/fonc.2020.591485

**Published:** 2021-01-08

**Authors:** Nan Hu, ShaoHan Yin, Qiwen Li, Haoqiang He, Linchang Zhong, Nan-Jie Gong, Jinyu Guo, Peiqiang Cai, Chuanmiao Xie, Hui Liu, Bo Qiu

**Affiliations:** ^1^ Department of Radiation Oncology, State Key Laboratory of Oncology in South China, Guangzhou, China; ^2^ Department of Radiation Oncology, Sun Yat-sen University Cancer Center, Guangzhou, China; ^3^ Department of Radiation Oncology, Guangdong Association Study of Thoracic Oncology, Guangzhou, China; ^4^ Department of Radiology, Sun Yat-sen University Cancer Center, Guangzhou, China; ^5^ Vector Lab for Intelligent Medical Imaging and Neural Engineering, International Innovation Center of Tsinghua University, Shanghai, China

**Keywords:** non-small cell lung cancer, MRI, heterogeneity, T stage, radiation therapy

## Abstract

**Objective:**

To improve the assessment of primary tumor heterogeneity in magnetic resonance imaging (MRI) of non-small cell lung cancer (NSCLC), we proposed a method using basic measurements from T1- and T2-weighted MRI.

**Methods:**

One hundred and four NSCLC patients with different T stages were studied. Fifty-two patients were analyzed as training group and another 52 as testing group. The ratios of standard deviation (SD)/mean signal value of primary tumor from T1-weighted (T1WI), T1-enhanced (T1C), T2-weighted (T2WI), and T2 fat suppression (T2fs) images were calculated. In the training group, correlation analyses were performed between the ratios and T stages. Then an ordinal regression model was built to generate the tumor heterogeneous index (THI) for evaluating the heterogeneity of tumor. The model was validated in the testing group.

**Results:**

There were 11, 32, 40, and 21 patients with T1, T2, T3, and T4 disease, respectively. In the training group, the median SD/mean on T1WI, T1C, T2WI, and T2fs sequences was 0.11, 0.19, 0.16, and 0.15 respectively. The SD/mean on T1C (p=0.003), T2WI (p=0.000), and T2fs sequences (p=0.002) correlated significantly with T stages. Patients with more advanced T stage showed higher SD/mean on T2-weighted, T2fs, and T1C sequences. The median THI in the training group was 2.15. THI correlated with T stage significantly (p=0.000). In the testing group, THI was also significantly related to T stages (p=0.001). Higher THI had relevance to more advanced T stage.

**Conclusions:**

The proposed ratio measurements and THI based on MRI can serve as functional radiomic markers that correlated with T stages for evaluating heterogeneity of lung tumors.

## Background

Malignant tumors usually consist of sub-clonal cells with different gene mutations, histology and morphology in a single lesion, which is termed as intratumoral heterogeneity (1–3). High level of heterogeneity has been reported to be associated with adverse survival outcomes in multiple cancer types (4, 5). Non-small cell lung cancer (NSCLC) is a highly heterogeneous disease regarding the genetic and phenotypic features (2, 3). The heterogeneity provides the fuel for drug resistance and treatment failure. The assessment of intratumoral heterogeneity helps in treatment decision and survival prediction. In patients with inoperable NSCLC, the diagnosis and treatment usually rely on a small amount of tissue by biopsy, which could not represent the chunk of tumor. Therefore, it’s important to develop a noninvasive method to evaluate the full spectrum of heterogeneity for primary lesions in NSCLC.

Intratumoral genetic heterogeneity leads to regional variety in stromal architecture, vascularity, glucose uptake, and water diffusion, which can be identified and quantified by medical imaging. Heterogeneity quantification by imaging has been reported to assist in distinctions on tumor types, grading, and different survival outcomes (6). Studies for lung cancer has primarily focused on computed tomography (CT) and positron emission tomography (PET) images. For example, texture analysis of computed tomography (CT) images in NSCLC have the potential to correlate with tumor hypoxia and angiogenesis (7). Intratumoral metabolic heterogeneity on ^18^F-FDG PET imaging has been shown to be associated with pathological type, differentiation, T stages, and recurrence in NSCLC (8, 9).

Magnetic resonance images (MRI) provides detailed anatomic information with high spatial contrast. The superior soft tissue resolution and lack of radiation make it a useful imaging modality for radiomic analysis. Advances in MR imaging technique, together with quantitative, and qualitative analysis, have expanded the role of MR imaging in lung cancer. The role of MRI in NSCLC has been investigated in multiple settings, including diagnosis, staging, response prediction and assessment, and postoperative lung function prediction (10–14). Moreover, MRI provides great insights into characterization of tumor heterogeneity (15, 16). Measuring heterogenous vascular features using dynamic contrast-enhanced (DCE) MRI and heterogenous cellular morphology using diffusion-weighted MRI (DWI) could yield important predictive biomarkers in lung cancer (16, 17). On the other hand, the assessment of intratumoral heterogeneity using routine MRI sequences has been less prominently studied in NSCLC (18), partly due to the variations in imaging protocol and acquisition signal. With the introduction of MR simulation and MR guidance into the radiation therapy workflow, the signal intensity analysis based on routine sequences become increasingly important and need further exploration.

We hypothesized that the texture features from MRI may be efficient in evaluating intratumoral heterogeneity. The aim of this study was to propose a method using basic texture measurements from T1- and T2-weighted MRI, which can improve the assessment of primary pulmonary tumor heterogeneity and provide more information on future MRI-guided radiation therapy.

## Materials and Methods

### Patients

From January 2016 to December 2018, 120 histologically diagnosed NSCLC patients with stage I–III disease who underwent radiation therapy in our center were included. Clinical data were collected from each patient including age, sex, histology, and tumor stage. Patients were staged based on the 8^th^ AJCC staging system for lung cancer. All patients had biopsy-approved pathological diagnosis of lung primary lesions. Chest MRI including unenhanced T2-weighted images (T2WI), T2WI with fat suppression, unenhanced/enhanced T1-weighted images (T1WI) had been acquired before radiation therapy. Informed consent was obtained from patients for the use of clinical and imaging data. This study was approved by institutional review board.

### MRI Acquisition

All MRI examinations were performed using the same 1.5 Tesla unit (GE Signa HDx 1.5; GE Healthcare, Milwaukee, Wisconsin, USA) with a combined eight channel phased-array surface coil. The following sequences were obtained for each patient: unenhanced T2WI in the coronal and axial planes; unenhanced T2WI with fat suppression in the axial planes; three-dimensional liver acquisition with volume acceleration (3D-LAVA) enhanced-scanning in the axial, sagittal, and coronal planes. The parameters of these sequences are listed in **Table 1**. For contrast enhancement, a 0.1 mmol/kg body weight bolus injection of gadopentetate dimeglumine was administered and the enhanced image was acquired ~25 s after the injection.

**Table 1:** Parameters for the magnetic resonance sequences.

Sequence botained	Scanning method	TR (ms)	TE (ms)	NEX	ST/spacing (mm)	FOV (cm)	Matrix
T2WI (sagittal plane)	FSE	>1,500	80	2	5/1	25	320×224
T2WI (axial plane)	FSE	≥2,000	85	2	3/1.5	25	320×224
FS T2WI (axial plane)	FSE	≥2,000	85	2	5/1	25	320×224
Enhanced-scanning (axial and coronal planes)	3D-LAVA	3–5	1–2.5	1	4/-2	25	512×224

T2WI, T2-weighted image; TR, repetition time; TE, echo time; NEX, nember of excitations; ST, slice thickness; FOV, field of view; FSE, fast spin-echo; SE, spin-echo; FS, fat suppression; SE-EPI, spin echo planar imaging.

### Image Processing and Analysis

**Figure 1:**
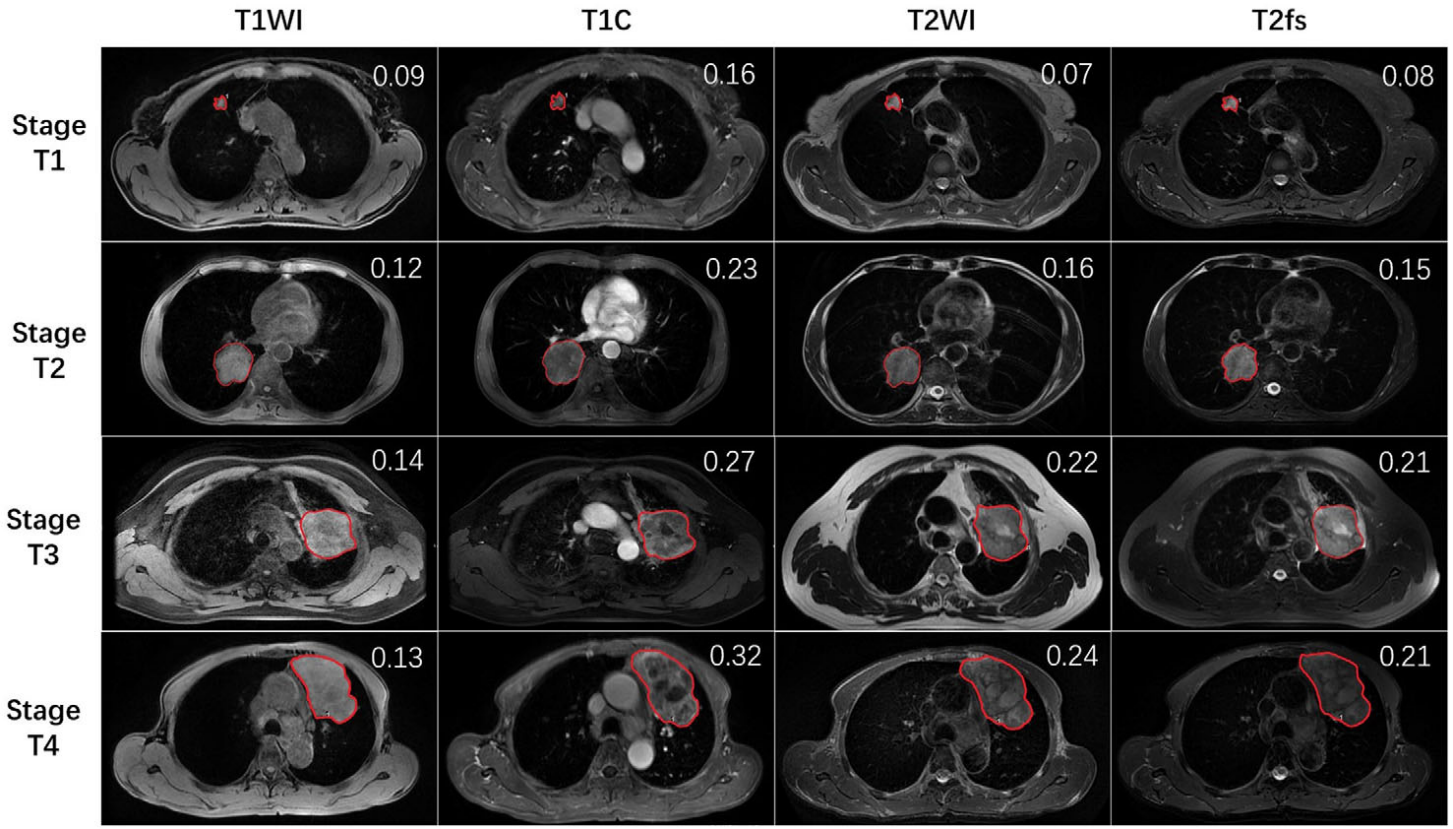
The representative MR images of stage T1–4 patients. Red lines delineate the ROI, which was delineated on the largest cross-sectional slice of primary lung tumor. The SD/mean value was shown on the right upper top of each image.

All MR images were viewed on a picture archiving and communication system workstation monitor (AW4.6; GE Healthcare, Milwaukee, Wisconsin, USA). The largest cross-sectional slice of primary tumor was selected. The region of interest (ROI) was manually contoured on T1WI, enhance T1 (T1C), T2WI, and T2 fat suppression (T2fs) images to encompass the entire cross-sectional area of the primary tumor (**Figure 1**). The encompassment of any adjacent normal lung tissue was avoided. Contouring was performed by one radiation oncologist and reviewed by a senior radiation oncologist and a radiologist as well. The mean and standard deviation (SD) of signal intensity of ROI was read from the GE workstation. To determine the intratumoral heterogeneity, the ratios between SD and mean value (SD/mean) were calculated. The higher the ratios value was, the higher the heterogeneity of the primary tumor, and vice versa.

### Statistical Analysis

We divided patients into the training and testing groups using propensity score matching (PSM) method with a caliper of 0.1 in a 1:1 ratio, with four covariates including sex, age, histology, and T stage. The SD/mean value from each sequence was presented as median and range, and compared using paired-sample t-test. In the training group, Spearman correlation analyses were performed to test the correlation between the SD/mean value and T stage. Variables with a p value <0.05 were selected into an ordinal logistic regression analysis. The ordinal logistic regression model is an extension of the binary model to the case of more than three outcomes which are naturally ordered. Then we generated a tumor heterogeneity index (THI) defined as the algebraic sum of imaging values in the model multiplied by their coefficients. The THI could be used to calculate the probability of each stage for an individual patient according to ordinal logistic regression equations. External validation of the model was performed in the testing group by calculating THI and the probability of T stage for each patient. Spearman correlation analyses were performed to test the correlation between the calculated THI and T stage. All statistical analyses were performed using SPSS ver. 24.0 software (IBM Corp., Armonk, NY), and differences were considered significant at a p-value < 0.05.

## Results

### Baseline Characteristics

A total of 104 of the included 120 consecutive patients were divided into the training and testing groups based on the above-mentioned PSM procedure. Patients in the training and testing groups were well matched with respect to age, sex, T stage, and histology (p>0.1) (**Table 2**). The demographic and clinical characteristics of all 104 patients were listed in **Table 2**. The median age was 59 years, ranging from 30 to 82. Eighty patients were male and 24 patients were female. The histology was squamous cell carcinoma in 62 patients and non-squamous cell carcinoma in 42 patients. Eleven, 32, 40, and 21 patients had stage T1, T2, T3, and T4 disease, respectively.

**Table 2:** The demographic and clinical characteristics of the 104 matched patients.

	Training group (n=52)	Testing group (n=52)	P value
Age (median, range)	58, 34~82	59, 30~78	0.985
Sex			0.642
Male	41	39	
Female	11	13	
Histology			0.424
Squamous	33	29	
Non-squamous	19	23	
T stage			0.779
T1	4	7	
T2	17	15	
T3	21	19	
T4	10	11	

Age, sex, histology, and T stage were well matched between training and testing group.

### The Heterogeneity of Primary Tumor on MRI

**Figure 2:**
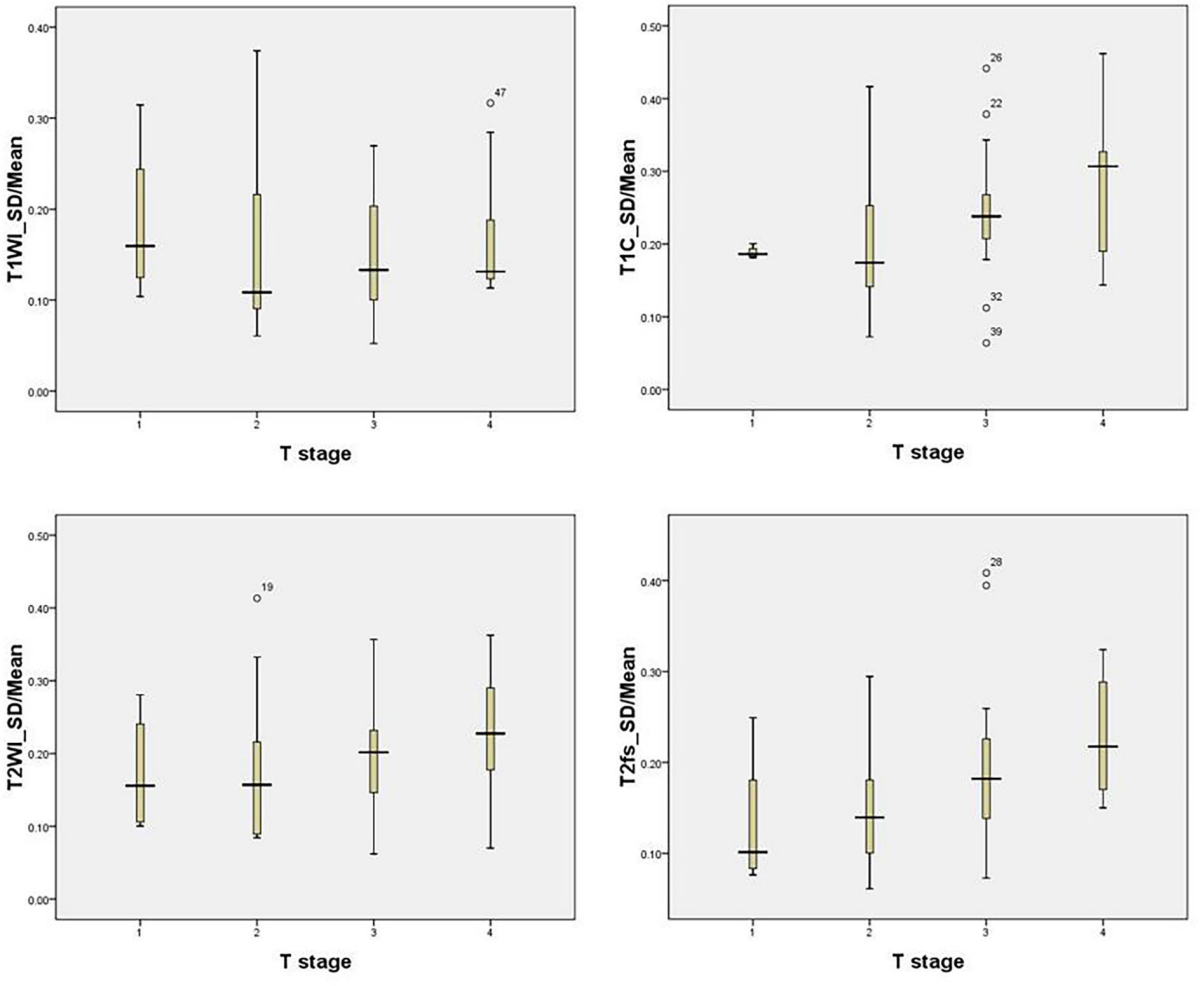
The SD/mean on T1WI, T1C, T2WI, and T2fs grouped by T stage in the training group. Patients with more advanced T stage showed higher SD/mean in T2-weighted, T2fs, and T1C sequences.

In the training group, the median SD/mean on T1WI, T1C, T2WI, and T2fs sequences was 0.11, 0.19, 0.16, and 0.15 respectively. The SD/mean was greatest on T1C, while smallest on T1WI. The SD/mean in T1C (p=0.003), T2WI (p=0.000), and T2fs sequences (p=0.002) correlated significantly with T stages. Patients with more advanced T stage showed higher SD/mean on T2WI, T2fs, and T1C sequences (**Figure 2**).

### The Development of Regression Model in the Training Group

In the training group, the SD/mean on T1C, T2WI, and T2fs sequences were entered into an ordinal logistic regression model to predict the T stage (**Table 3**). The model fitting information indicated a significance of 0.000. The SD/mean on T2fs (p=0.017) and T1C (p=0.043) were independently predictive of T stage.

**Table 3:** Ordinal logistic regression analysis.

	Coefficient	P value	OR
Threshold[T = 1]	−0.453	.604	—
Threshold[T = 2]	1.924	.022	—
Threshold[T = 3]	4.159	.000	—
SD/mean_T2WI	−11.301	.077	0.462
SD/mean_T2fs	14.906	.017	4.428
SD/mean_T1C	7.748	.043	0.744

The SD/mean on T1C, T2WI, and T2fs sequences were entered into an ordinal logistic regression model to predict the T stage. The model fitting information indicated a significance of 0.000.

Based on the model, a tumor heterogeneous index (THI) that consists of these three variates was developed as the following equation:

THI=7.748×SD/mean_T1C−11.301×SD/mean_T2+14.906×SD/mean_T2fs

The median THI in the training group was 2.15 (range 0.81~4.85). THI correlated with T stage significantly (p=0.000).

The probability of any T stage for each patient could be calculated by the ordinal logistic regression rule:

T1=P(Y≤1)=1/(1+exp[−(−0.453−TH1)])

T2=P(Y≤2)−P(Y≤1)=1/(1+exp[−(1.924−TH1)])−1/(1+exp[−(0.453−TH1)])

T3=P(Y≤3)−P(Y/≤2)=1/(1+exp[−(4.159−TH1)])−1/(1+exp[−(1.924−TH1)])

T4=1−P(Y≤3)=1−1/(1+exp[−(5.140−TH1)])

### Validation of the Model and Tumor Heterogeneity Index in the Testing Group

**Figure 3:**
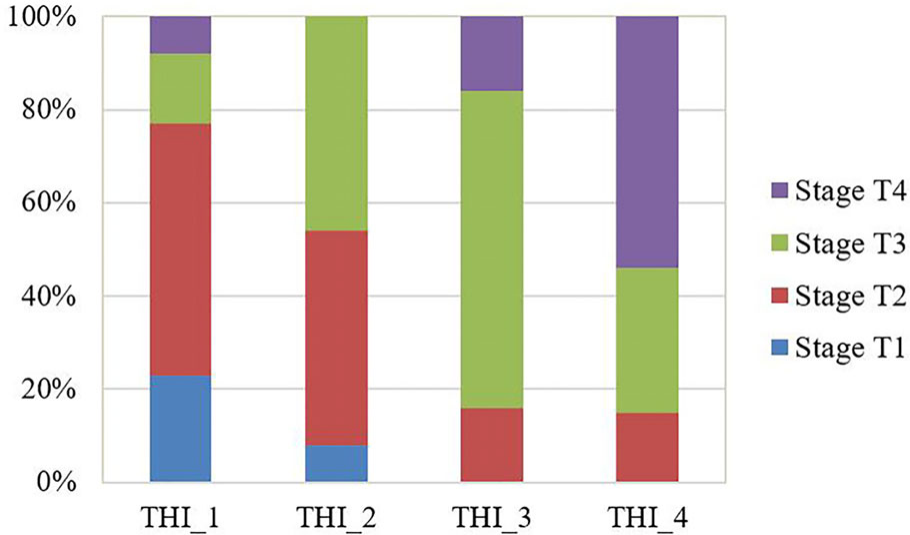
The observed proportion of T stages in the testing group stratified by the tumor heterogeneity index (THI). THI was categorized by quantile to THI_1: 0.33~1.42; THI_2: 1.44~2.04; THI_3: 2.09~2.60; THI_4: 2.67~5.06. The proportion of more advanced stages grew gradually as the THI increased.

THI had been calculated for each patient in the testing group according to the above equation. The median THI was 2.06 (range, 0.33~5.06). THI correlated with T stage significantly (p=0.001). The proportion of more advanced stages grew gradually as the THI increased in the testing group (**Figure 3**).

The probability of each stage was calculated for individual patient according to the above equations to generated the estimated T stage. The actual and estimated T stage were listed in **Table 4**. The model predicted T stage accurately in 61.5% (32/52) of patients.

**Table 4:** The actual and estimated T stages in the testing group.

Actual T stage	Estimated T stage				
	1	2	3	4	Total
1	**5**	2	0	0	7
2	3	**10**	2	0	15
3	0	3	**10**	6	19
4	0	1	3	**7**	11

The bold values are the number of cases in which the predicted T stage matches the actual T stage successfully.

## Discussion

Intratumoral heterogeneity is an important feature of malignant tumors. There has been considerable effect to use medical imaging to depicts spatial heterogeneity in tumors (6). The advantage of MRI compared with other imaging approach, such as CT and PET, resides in its potential to provide a multi-parameter sequences (T1WI, T2WI, diffusion-weighted, flow-weighted, etc.) at a high spatial resolution. In NSCLC, the signal intensity of primary lesion on MRI is often complexed by fibrous tissue signal, necrotic signal, septations, and vascular void signal. In this study, we quantified the signal heterogeneity on T1WI, T1C, T2WI, and T2fs sequences by measurement of SD/Mean. The signal heterogeneity on T1C, T2WI, and T2fs sequences correlated with T stages significantly. A model had been developed based on the measurements to predict T stages. Then the tumor heterogeneity index (THI) was generated which could be used as a potential radiomic marker for MRI-guided diagnosis and radiotherapy on NSCLC.

Our results showed higher THI was associated with more advanced T stages. It was in accordance with the finding that the metabolic heterogeneity of primary tumor from ^18^F-FDG PET showed a stepwise increase with the increase of T stages in NSCLC (8). As T stage is a well-established prognostic factor, imaging heterogeneity has been regarded as a potent biomarker for prognosis as well. The intratumor heterogeneity is a distinct predictive factor of response to radiation therapy, mainly due to the resistant subpopulations of cells (19). Non-enhancing tumor fraction assessed by DCE MRI subtraction was found to be a predictor of decrease in tumor volume in response to chemoradiotherapy (20). Texture features derived from MRI was reported to effectively predict tumor response after radiotherapy (18). Taken together, these findings implied that the heterogeneity assessed by MRI reflected the biological behaviors of tumors thus to provide important biological information for tumor diagnosis and treatment in NSCLC.

Although MRI emerges as an effective method for assessing tumor heterogeneity, the research of optimal sequences or method for quantifying heterogeneity are still processing. At present, studies on heterogeneity assessment by MRI mostly focus on DCE analysis in NSCLC. The semiquantitative perfusion, histogram, and texture parameters from DCE were shown to be prognostic of clinical outcomes (17, 21, 22). However, compared with routine sequences, DCE was not conveniently available in routine practice and relies on post-processing software for data analysis. Therefore, we explored the role of routine sequences in the assessment of intratumoral heterogeneity in NSCLC, which might provide practical information on MRI-guided diagnosis and radiation therapy (MRI simulation and MRI Linac). In our study, tumor on T1C showed the most remarkable signal heterogeneity among the four routine sequences. While unenhanced T1 sequence exhibited the most homogenous imaging. It may be expected that unenhanced images could not well differentiate regions of viable tumor, hypoxic tissues, necrosis or myxoid changes. Contrast-enhanced imaging allows for visualization of more diverse intratumoral components with heterogeneity in enhancement, which can be attributed to heterogeneity of intratumoral perfusion and permeability (23). Therefore, T1C might provide more spatially rich information, and be an important sequence for MRI-based radiomic analysis for intratumoral heterogeneity.

**Figure 4:**
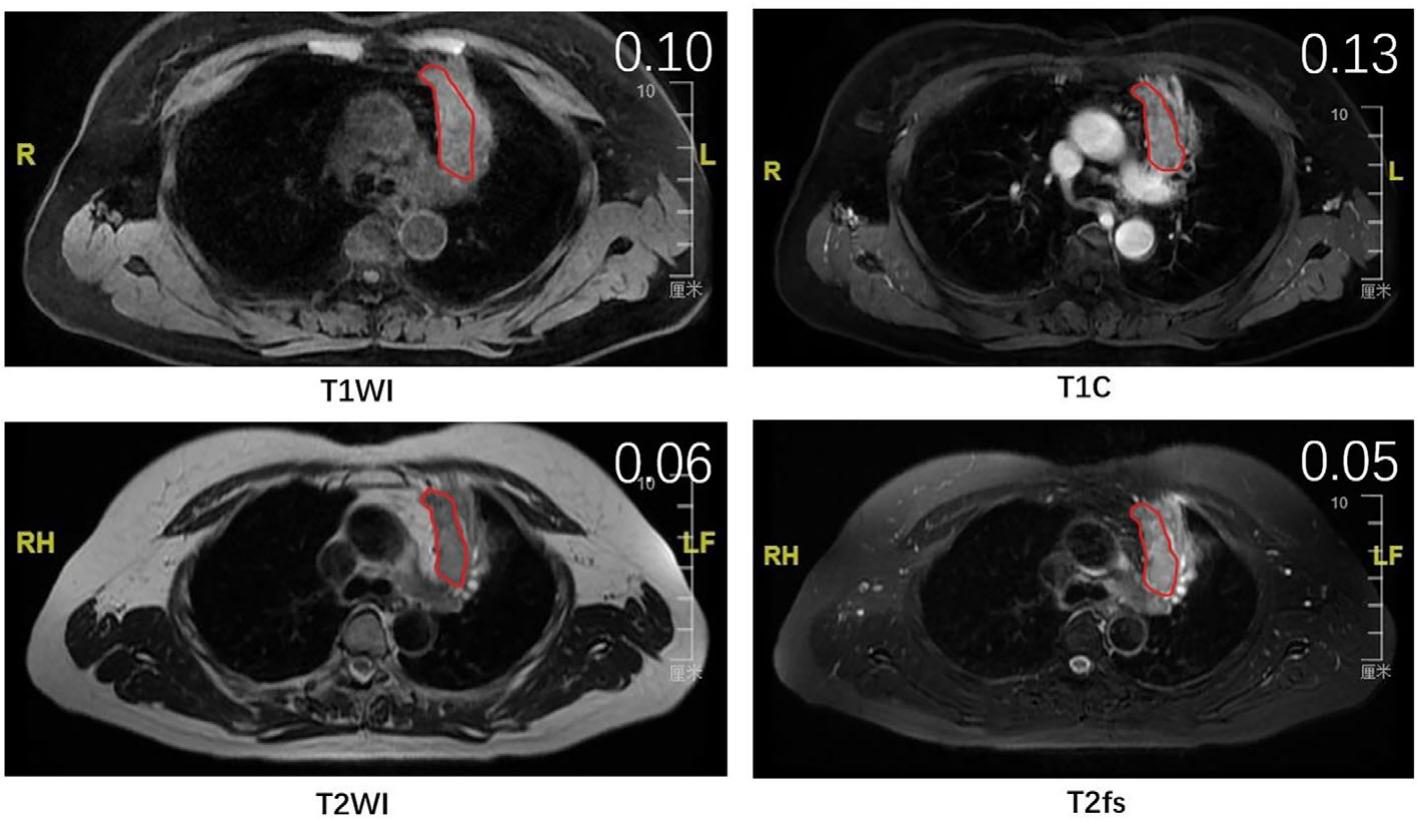
The MR images of a patient with stage T4 that was falsely predicted as stage T2 by the model. Red lines delineate the ROI, which was delineated on the largest cross-sectional slice of primary lung tumor. The SD/mean value was shown on the right upper top of each image.

Other than T1C, the heterogeneity measurements from T2WI and T2fs also correlated with T stages. Combining analysis of multiple sequences might provide a more significant marker than single sequence alone. Therefore, we generated a model based on signal heterogeneity from T1C, T2WI, T2fs to predict T stages. By external validation, this model predicted T stages with an accuracy of nearly 62% in the testing group. It is noteworthy that more than 40% of patients were falsely predicted in the testing group. This indicated that a model derived from merely MRI parameters could not yield a satisfactory performance in predicting T stages. Other factors, such as the location of primary tumor and the presence of atelectasis or obstructive pneumonitis, might also determine the T stages. For instance, a primary tumor of small size that invades mediastinum is staged as T4, while it might present with relatively homogenous appearance and therefore is predicted as T2 according to the model (**Figure 4**). Despite the suboptimal performance, it certainly added value in predictive models incorporating multiple clinical and imaging variables. From this point of view, we generated a novel heterogeneity score from the regression model, term as tumor heterogeneity index (THI), defined as the algebraic sum of the heterogeneity measurements from three sequences multiplied by their coefficients. THI represented the level of heterogeneity, which significantly correlated with T stages in the training and testing groups. Higher THI had relevance to more advanced T stage and possibly worse prognosis. THI could be easily accessed from routine MRI imaging, and therefore conveniently serves as an efficient MRI biomarker.

Besides quantifying the level of heterogeneity, another purpose of heterogeneity analysis is to identify the subregions with different biology that respond differentially to treatment. Kim *et al*. used clustering of PET and DWI to identify highly aggressive subregions in NSCLC. They found the volume of subregion with high aggressiveness was a negative prognostic factor of survival (16). Radiation therapy is the backbone of treatment for locally advanced NSCLC. High-dose radiation was associated with improved local-regional control. However, escalating the radiation dose to the whole tumor volume is limited by normal tissue toxicity. Nowadays, radiation planning using intensity modulated technique allows for different dose distributions inside a tumor volume. Therefore, improvement of local control could be achieved by taking into account intratumoral heterogeneity and delivering higher dose to resistant subregions (24). Kong *et al*. adapted the target volume based on mid-treatment PET and delivered higher-dose radiation to the FDG-avid areas of the tumor, which achieved favorable local-regional control. Compared with PET, MRI could be conveniently acquired before and during the course of radiation. MRI-guided radiotherapy with hybrid MR linear accelerator creates new perspectives towards an individualized planning and treatment approach (25). Therefore, fully depicting the intratumoral heterogeneity on MRI will help identify the resistant subregions and provide evidence for adaptation strategies.

This study has several limitations. Firstly, we quantified the parameters from the largest cross-sectional slice instead of the whole tumor volume. Although tumor heterogeneity measured by the two methods was similar (26), analysis of the whole tumor can theoretically capture more heterogeneous internal components. Secondly, in comparison with CT or PET, scanner and sequence acquisition parameters of MRI have great influence on signal intensity measurements and heterogeneity quantification. Therefore, in order to minimize the influence, we scanned all patients in the same scanner with the same parameters.

## Conclusions

The aim of this study was to propose a method using basic texture measurements from T1- and T2-weighted MRI which can improve the assessment of primary pulmonary tumor heterogeneity and biological behaviors. We found the signal heterogeneity on T1C, T2WI, and T2fs sequences, in terms of SD/mean, correlated positively with T stages. The proposed ratio measurements and THI based on clinical routine MR images can serve as functional radiomic markers that correlated with T stages for evaluating heterogeneity of lung tumors, and provide more information on future MRI-guided radiation therapy. Further studies are warranted to validate the role of THI in response and survival prediction.

## Data Availability Statement

The original contributions presented in the study are included in the article/supplementary material. Further inquiries can be directed to the corresponding authors.

## Ethics Statement

The studies involving human participants were reviewed and approved by Ethics Committee of cancer center of Sun Yat sen University. Written informed consent for participation was not required for this study in accordance with the national legislation and the institutional requirements. Written informed consent was obtained from the individual(s) for the publication of any potentially identifiable images or data included in this article.

## Author Contributions

BQ and HL are the main leaders of the project, making major contributions to the project design and article writing. NH is responsible for the main data statistics, image processing, and article writing. QL is responsible for part of the data statistics and image processing work. JG is responsible for data statistics. HH, SY, LZ, NG-J, PC, and CX helped with image processing and analysis. All authors contributed to the article and approved the submitted version.

## Conflict of Interest

Author NG-J was employed by the company Shanghai United Imaging Healthcare Co., Ltd.

The remaining authors declare that the research was conducted in the absence of any commercial or financial relationships that could be construed as a potential conflict of interest.
